# Underwater Acoustic Wireless Sensor Networks: Advances and Future Trends in Physical, MAC and Routing Layers

**DOI:** 10.3390/s140100795

**Published:** 2014-01-06

**Authors:** Salvador Climent, Antonio Sanchez, Juan Vicente Capella, Nirvana Meratnia, Juan Jose Serrano

**Affiliations:** 1 Institut ITACA, Universitat Politècnica de València, Edifici 8G, València 46022, Spain; E-Mails: ansanma7@itaca.upv.es (A.S.); jcapella@disca.upv.es (J.V.C.); jserrano@itaca.upv.es (J.J.S.); 2 Pervasive Systems, University of Twente, P. O. Box 217, Enschede 7500 AE, The Netherlands; E-Mail: n.meratnia@utwente.nl

**Keywords:** underwater sensor networks, survey, future trends, physical layer, medium access control layer, routing layer, security

## Abstract

This survey aims to provide a comprehensive overview of the current research on underwater wireless sensor networks, focusing on the lower layers of the communication stack, and envisions future trends and challenges. It analyzes the current state-of-the-art on the physical, medium access control and routing layers. It summarizes their security threads and surveys the currently proposed studies. Current envisioned niches for further advances in underwater networks research range from efficient, low-power algorithms and modulations to intelligent, energy-aware routing and medium access control protocols.

## 1. Introduction

Underwater acoustic communication has been under research for at least half a century now. One of the first underwater communication devices was the underwater phone, developed for the US Navy after World War II [1].

During the last decade, this form of communication has received increasing attention, owing to its many scientific, military and commercial applications. These applications range from tactical surveillance to the study of marine life and include unmanned vehicle communication, pollution monitoring, oil extraction monitoring and aquiculture monitoring.

Electromagnetic waves, optical waves and acoustic waves have been successfully used in underwater wireless sensor networks (UWSNs) [2,3]. Nevertheless, radio frequency (RF) waves are affected by high attenuation in water (especially at higher frequencies) [2], thus requiring high transmission power and large antennae [4]. Optical waves can be used to achieve ultra-high-data-rate communications (Gbit/s), but are rapidly scattered and absorbed in water, so they are only reliable for short-distance links [2].

In contrast, acoustic waves enable communications over long-range links, because they suffer from relatively low absorption. Thus, this is the preferred technology to develop reliable UWSNs [3] and is the main focus of this paper. In [5], the characteristics of the acoustic underwater channel and the difficulties in underwater communication are discussed. The differences between acoustic and radio-based communication open a new research field in UWSNs.

Despite the increased complexity of acoustic transmissions when compared to RF transmissions, researchers have made enormous advances since the underwater telephone. Nowadays, commercially available underwater modems are able to transmit up to 30 kbps over distances ranging from a hundred meters to a few kilometers [6,7].

Given these advances in underwater transmission capabilities, an increasing amount of research has been focused on building networks of underwater nodes. Given the long propagation delays, direct use of medium access control (MAC) and routing protocols of previously existing RF networks is not advisable. Hence, a great deal of research has been focused on this issue. Moreover, some of these protocols require time synchronization and localization. These problems must be revisited, because propagation time is not usually taken into account in RF networks.

This survey aims to provide a comprehensive overview of the current research on underwater wireless sensor networks, focusing on the lower layers of the communication stack, and envisions future trends and challenges. It analyzes the current state-of-the-art for the physical (Section 2), MAC (Section 3) and routing layers (Section 4). In addition, it summarizes their security threads and surveys the currently proposed studies. Finally, conclusions on the current state-of-the-art in UWSNs and future challenges to be faced are presented.

## 2. Modem Physical Layer

The physical layer defines the mechanism for transmitting bits over a physical link channel connecting network nodes. The transmitter converts bit streams into a physical signal that is propagated through the physical layer. On the other side, the receiver should be able to reverse the process and provide the original bit stream to the upper communication layers.

The main issues tackled by the physical layer in a UWSN and discussed throughout this section are as follows: interface to physical transmission media, modulation, equalization filtering, efficient carrier sense and collision detection (used by the MAC layer); additionally, other essential services include bit rate, bit synchronization and forward error correction.

The goal of physical layer design is to provide robust and reliable communications with the highest spectrum efficiency, which is the ratio of the communication bit rate to the channel bandwidth. Additionally, because UWSN nodes are usually autonomous and powered by batteries, power saving is also a key physical layer issue.

### 2.1. Underwater Acoustic Channel Characterization

#### 2.1.1. Attenuation and Noise

The path loss of the signal can be modeled as Equation (1), where *f* is the signal frequency and *l* is the transmission distance. The path-loss component, *k*, models the spreading loss and is usually between one and two. The absorption coefficient can be obtained using Thorp empirical equation, as described in [8], and is expressed in Equation (2).

(1) $$A(l,f)=l^{k}a{\left(f\right)}^{l}$$

(2) $$10\log a(f)=\frac{0.11f^{2}}{\left(1+f^{2}\right)}+\frac{44f^{2}}{\left(4,100+f^{2}\right)}+0.000275f^{2}+0.0003$$

The ambient noise (*N*) is dependent on the deployment environment. For ocean environments, empirical formulas exist that model the noise from four sources: turbulence (*N_t_*), shipping (*N_s_*), waves (*N_w_*) and thermal noise (*N_th_*) [9]. Formula Equation (3) gives the power spectral density of the four noise components in decibels re*µ* Pa per Hz as a function of frequency (*f*) in kHz. In addition, *s* is the shipping factor, which ranges from zero to one for low and high activities, respectively, and *w* is the wind speed in *m*/*s*:

(3) $$\begin{array}{ll} 10\log N_{t}(f)= & 17-30\log f\\ 10\log N_{s}(f)= & 40+20(s-0.5)+26\log(f)-60\log(f+0.03)\\ 10\log N_{w}(f)= & 50+7.5w^{1/2}+20\log f-40\log(f+0.4)\\ 10\log N_{th}(f)= & -15+20\log f\\ N(f)= & N_{t}(f)+N_{s}(f)+N_{w}(f)+N_{th}(f) \end{array}$$

**Figure 1.**
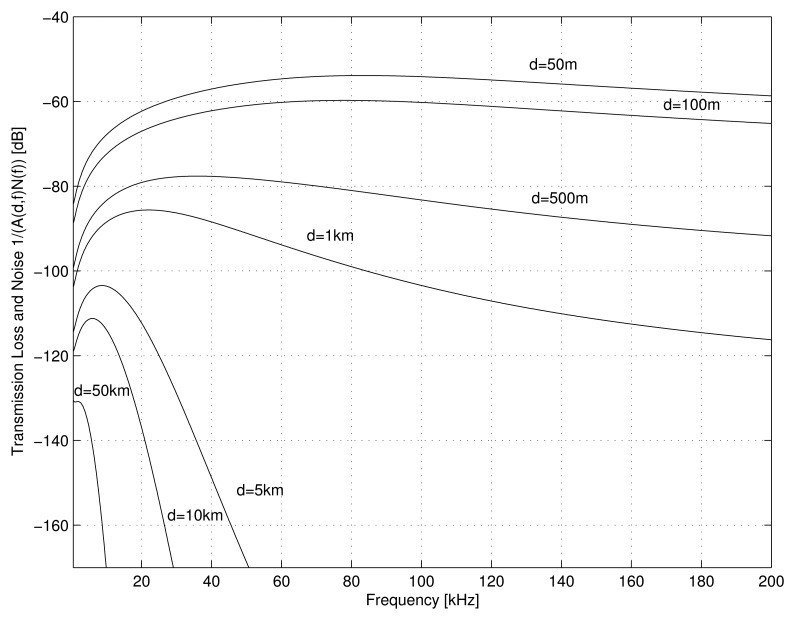
Narrow-band signal-to-noise ratio (SNR; 1/(*AN*)) as a function of frequency for varying transmission distance. Environmental factors used: practical spreading, *k* = 1.5; wind speed, *w* = 3 m/s; and moderate shipping activity, *s* = 0.5.

As introduced in previous works [3,10], the overall effect of the transmission loss (*A*(*l*, *f*)) and the noise density (*N*(*f*)) are evaluated as the narrow-band signal-to-noise ratio (SNR) for different distances and different frequencies. The results shown in Figure 1 are useful for estimating the optimal frequency band, attending to the area that the network should cover. For example, larger areas should be covered using low-frequency acoustics (below 10 kHz, as expressed in [3]), but for medium or short distances in high-density networks, frequencies around 80 kHz are preferred.

#### 2.1.2. Multipath

Multipath in underwater channels is mainly caused by two relevant factors: wave reflection at the surface, bottom and any object and sound refraction in the water [5].

The reflection coefficient is equal to −1 under ideal conditions, while bottom reflection coefficients depend on the type of bottom (hard, soft) and the grazing angle [5].

The second effect is a consequence of Snell's law. The propagation speed of an acoustic signal underwater is, on average, approximately $1,500\;\frac{m}{s}$; however, the actual value depends on the salinity, temperature and pressure of the medium, among other factors. A complete nine-term equation for calculating propagation speed is defined in [11], as shown in Equation (4), where *T* denotes the temperature in degrees Celsius, *D*, the depth in meters, and *S*, the salinity in parts per thousand of the water.

(4) $$\begin{array}{r} c=1448.96+4.591T-5.304\times 10^{-2}T^{2}+2.374\times 10^{-4}T^{3}\\ +1.340(S-35)+1.630\times 10^{-2}D+1.675\times 10^{-7}D^{2}\\ -1.025\times 10^{-2}T(S-35)-7.139\times 10^{-13}D^{3} \end{array}$$

**Figure 2.**
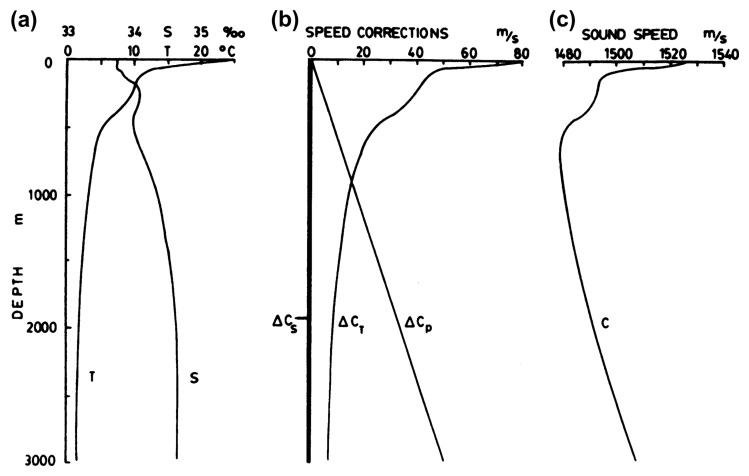
Temperature, salinity data and their impact on the sound speed profile. Data retrieved from the Papa station placed in the Pacific ocean (39°N, 146°W) in August, 1959.

Figure 2 shows an example of how sound speed propagation changes along a water column placed in the Pacific ocean. As can be seen in subfigure (a), the speed rapidly decreases until approximately a 600-m depth, remaining nearly constant around 4 in deep oceans waters. In subfigure (b), where the individual contributions of temperature, salinity and pressure to sound speed are displayed, shows that deep water sound speed variation mainly depends on the increasing pressure.

**Figure 3.**
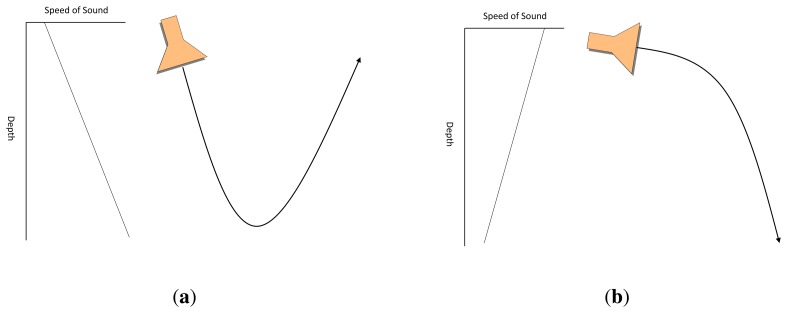
Acoustic Ray Tracing for Sound Speed Profiles with Positive and Negative Gradients. (**a**) Positive Gradient Sound Speed Profile. (**b**) Negative Gradient Sound Speed Profile.

Combination of reflection and refraction can lead to some remarkable effects, as explained for the sonar propagation in [12]:

- Positive temperature gradient combined with surface reflection. Due to positive gradient, vertical rays are deflected upwards, as shown in Figure 3a. Both deflected and direct rays are reflected on the water surface. Reflected rays are once again deflected, and so on. As a result, acoustic waves barely achieve depth areas under these conditions.
- Combination of positive and negative temperature gradient. Near the surface, vertical rays are deflected upwards as in the previous case. However, acoustic waves that reach negative temperature gradient area are deflected downwards as shown in Figure 3b. As a consequence, there is some area -close to the temperature gradient discontinuity region- in which received acoustic wave intensity is very low.
- Combination of negative and positive temperature gradient. In contrast to the previous case, this combination leads to a concentration of the rays around the gradient discontinuity region. This region is known as the SOFAR channel and it can be used to transmit acoustic waves to long distances.

Besides, conditions change continuously in both long and short time-scales, e.g., temperature variation and surface waves, respectively. The last effect is especially critical, since acoustic reflection points are continuously displaced, resulting in scattering and Doppler spreading, due to the changing path length [5].

As a conclusion, the impulse response of an acoustic channel is strongly correlated to the deployment scenario: holography, depth, particular environmental conditions, final nodes placement and even the current time slot have a severe impact on the channel response.

#### 2.1.3. Doppler Effect

The relative motion of the transmitter and the receiver produces a Doppler effect, which causes an additional signal distortion. The Doppler effect is significantly important in networks, including mobile elements, such as autonomous underwater vehicles (AUV).

As described in [13], Doppler-shift can be seen as either a frequency shift or a time scaling (expansion/compression) of the signal waveform. These effects are shown in Equation (5), where s(t) and r(t) are the source and the received signal, respectively. These effects are also reflected in a frequency shift of the received signal following Equation (6). The distortion factor, Δ, is proportional to the modem's relative speed, as shown in Equation (7)

(5) r(t)=s((1+Δ)t)

(6) $$f_{r}=(1+\Delta )f_{s}$$

(7) $$\Delta =\frac{\upsilon_{s,r}}{c}$$

#### 2.1.4. Channel Propagation Models

In conclusion, both frequency-dependent absorption and noise and low and variable propagation speed have a significant impact on the design of underwater communication networks. It has been demonstrated that different scenario characteristics and even current different time spots lead to different channel responses.

Simulations are a very convenient way of testing algorithms and protocols before their actual deployment. However, one has to be certain that the simulation results are as accurate as possible. The use of, for example, a too simple of a physical model can alter the behavior and, sometimes, lead to wrong or incomplete conclusions [14,15].

The most simple propagation model is the empirical Thorp's formula presented in Equation (2). Although the simulation can be very fast, attenuation formula accuracy is very poor, as long as it is only based on the frequency and the distance from the nodes, ignoring other important physical criteria, such as ocean waves, depth, holography, *etc*.

The next model in complexity is the Monterrey Miami Parabolic Equation (MMPE) [16], implemented in OPNETin a simulator called Xie Gibson [17]. Its main equation is shown in Equation (8) This model offers a better description of the attenuation calculation by including the effects of ocean waves, the depth of the nodes and the seafloor multipath. However, several parameters need to be computed before running any simulation [18].

(8) $$PL(t)=m(f,s,d_{S},d_{R})+w(t)+e()$$

where:

- *PL*(*t*) : propagation loss from node S to node R.
- *m*() : propagation loss without random and periodic components.
- *f* : signal frequency (kHz).
- *d_S_* : sender's depth (meters).
- *d_R_* : receiver's depth (meters).
- *s* : distance between S and R (meters).
- *w*(*t*) : function to approximate periodic signal loss due to wave movement.
- *e*() : signal loss due to random noise or error.

Last, some models are based on the solution of ray equations, described in detail in [19], to determine the ray coordinates of the acoustic signal propagation. Recent simulators, such as the World Ocean Simulator System [20] in Network Simulator 2, and some interesting OPNET simulator extensions [18] incorporate this model. Several woks have been performed to validate this model with real measurements [20–26]. Integrating empirical data from world databases that measure the sound speed profile, bathymetry, floor sediment and ocean wave motion, this model shows a behavior that is very close to reality.

Recently, some extensions of the previous models have been developed to simulate sea surface dynamics [27], variable attenuation and the Doppler effect produced, as described in Section 2.1.2. These models use successive runs of the propagation model as the surface evolves, so simulation performance decreases significantly.

### 2.2. Modulation

By modulating the signal, one or more properties (amplitude, phase or frequency) of a periodic waveform (carrier signals) are changed according to another signal (modulating signal) that contains the information to be transferred. Using this technique, the modulating signal spectrum is matched to the current communication channel characteristics.

As previously described, maximum spectral efficiency is mandatory to exploit the limited bandwidth available in the underwater acoustic channel, and, in addition, energy constraints must be taken into account. Hence, several research works were focused on finding the optimal modulation scheme.

#### 2.2.1. Non-Coherent Modulation

At first, works on acoustic communications were mainly focused on non-coherent modulation methods. In particular, on-off keying (OOK) [28,29] and frequency-shift keying (FSK) [2,30–32] modulation schemes based on energy detection were favored, because decoding does not require carrier-phase tracking. The main advantages of these modulations are their reliability and simplicity, so modems do not need high-resource processors with higher power consumption [33]. However, spectral efficiency using these methods is very low, because of inter-symbol and inter-carrier interference generated by Doppler and multipath spread.

#### 2.2.2. Coherent Modulation

To increase the spectral efficiency and communication range, researchers have explored different alternatives based on phase-coherent modulation techniques, such as phase-shift keying (PSK) [34] and quadrature amplitude modulation (QAM) [35]. However, in order to face acoustic channel spread without significant spectrum efficiency loss, incoming signals should be equalized according to the channel response and decoded later. By using these techniques, modem complexity and power consumption increase.

#### 2.2.3. Special Modulation Schemes

Different advanced modulations are being used in order to try to better utilize the available bandwidth. In direct-sequence spread-spectrum (DSSS) modulation, the original signal is spread over a wider spectrum in a ratio of $L=\frac{W}{B}$(B is the original signal bandwidth, and W is the spread signal bandwidth). The original signal is multiplied by a pseudo-noise (PN) code with length L. At the receiver side, the received signal is de-spread using the same spreading code before demodulation [36]. Multi-user communication may be supported by assigning each user with a unique spreading sequence with good autocorrelation and cross-correlation properties that can resist interference from multiple users [37]. Moreover, fading effects are also corrected if a RAKEreceiver is employed. Nevertheless, the main drawback of this technique is the bandwidth efficiency, which is lower than 0.5 bit/s/Hz.

Long delay spread can be reduced by using multiple-carrier modulations [38,39]. A certain number of closely-spaced orthogonal sub-carrier signals are used to carry data on several parallel data streams or channels, using a conventional modulation scheme. Thus, each sub-carrier can be considered as a flat-fading sub-channel that is easy to equalize by multiplying it by a single complex value. The most well-known example is orthogonal frequency-division multiplexing (OFDM).

Another interesting approach to avoid multipath spread is the use of multiple transmitters and receivers to take advantage of spatial diversity. In this technique, multiple copies of the same information are sent through different independently fading channels, which increase the probability of correct reception. On the other hand, transmitting multiple independent streams of information through spatial channels may increase the total available data rate. The resulting system is labeled as a multiple-input-multiple-output (MIMO) system [40,41].

### 2.3. Acoustic Channel Equalization

Because the acoustic channel is bandwidth limited, it distorts the transmitted signal, causing the transmitted symbol to spread to adjacent symbols. This interference is known as inter-symbol interference (ISI). Equalization is the process of filtering the received signal to cancel the ISI introduced by the channel impulse response.

In addition, the inter-symbol interface caused by multipath can be avoided by inserting guard times between successive symbols [42]. Different echoes of the transmitted signal arrive at different times with different delays, and the modem should still be able to successfully decode the message. Moreover, to avoid multipath frequency spread, guard frequency bands are also needed [42]. These techniques are suitable for non-coherent communications, where no phase tracking is required. In real applications, spectrum efficiency is very low, because the data rate decreases, and higher bandwidth is needed to insert these guards.

In coherent communications, another approach is explored. Conventional equalization algorithms require the transmission of a training data sequence to enable the receiver to estimate the channel. An adaptive decision feedback equalizer (DFE) can be used to compensate for the effects of multipath [43]. One of the drawbacks of DFE is that errors may propagate, because of wrong decisions fed into the feedback loop. Strong forward error correction (FEC) codes may be used to combat the error propagation and, as a result, reduce the bit error rate (BER) [10].

As already described, RAKE filters used in DSSS, multi-carrier modulation and MIMO systems are also oriented towards solving the channel spread cancellation problem, because it is an essential issue that must be tackled in acoustic communications.

The experiments run by the University of Birmingham and Newcastle, U.K., show that bit error rate (BER) can be reduced from 10^−2^, without any equalization, up to 10^−3^ using MIMO and LMStechniques, respectively [44].

### 2.4. Acoustic Triggered Wake-Up Systems

In normal operation, UWSN nodes alternate data communication and IDLEmodes. In an ideal scenario, nodes remain switched off, as long as no communication is requested, so minimal energy is consumed. However, synchronization based on clock sharing strategies presents an additional energy waste to the minimum required to transmit or receive [45]. Moreover, in an event-driven communication, which starts when some specific event is recognized, the wireless interface tends to remain active longer than is necessary to complete a packet transmission.

The UWSN can save significant power when an acoustic-triggered wake-up (AT-WU) system is used. Using this technique, a node is able to react to certain acoustic signals, reactivating the node if needed. However, it requires specific hardware to perform this task. The results of simulations of simple link [46] and carrier sense multiple access (CSMA) MAC networks [47] are close to the optimal scenario. It is crucial that this new hardware dissipates ultra-low power, as it always remains active.

Very little work has been done on asynchronous AT-WU, though there are various state-of-the-art commercial modems that implement this technology. The main references so far to the research of acoustic modems enhanced with AT-WU are the Wills modem [30] and ITACAmodem [48]. The power consumptions of these modems when waiting for an acoustic wake-up signal are 500 µW and 10 µW, respectively.

### 2.5. Commercial and Research Modems

In contrast to RF wireless sensor networks, only a few companies are engaged in developing modems for underwater sensor networks. The most reliable modems are shown in Table 1. The prices of the listed modems are above $1,000 [2], which makes the deployment of dense sensor networks similar to terrestrial networks very expensive.

These modems have been included in this study, because they are the result of modern ongoing research, with several publications in relevant international conferences [34,49,50]. Moreover, these companies have collaborated with underwater researchers in recent marine projects, such as: European FP5, FP6 and FP7 in the case of Aquatec [51]; ONRU.S. projects in the case of WHOI [52]; and private contractors in the case of Teledyne Benthos [7].

In order to achieve a desirable trade-off between the different parameters compared in Table 1 and decrease costs, research is still in progress on acoustic modems. In Table 2, a comparison of state-of-the-art research modems is presented. Unlike commercial modems, comparisons among research modems are significantly more complicated, as the information provided in different papers is less standardized. In this case, the center frequency, data rate and modulation are detailed.

The performances of the different modulations are also compared in Table 2. Although more complex modulations (DSSS and OFDM) yield promising communication performance results, simple modulations (FSK and OOK) are also used in several reliable recent works, but with low communication performance.

### 2.6. Security at the Physical Layer

A jamming attack is a type of denial-of-service attack that consists of interfering by continuously transmitting on the same wireless channel neighboring nodes use to communicate. It is a common well-known attack in wireless networks and some solutions, like frequency hopping and spread spectrum, can be applied to mitigate this attack [57]. Zuba *et al.* perform in [58] a real-worldtest to introduce various types of jamming attacks on different underwater modems. However, further research needs to be conducted for UWSNs, due to their special features.

### 2.7. Conclusions and Future Challenges

In UWSNs, energy consumption can be argued to be the largest concern. Because nodes are battery powered, energy efficiency at the physical layer is mandatory. However, at the same time, it is desirable to reach further transmission distances and to achieve better bandwidth usage. Hence, further research should be conducted into developing energy-aware techniques that will make more efficient use of the available bandwidth. On the one hand, effective bandwidth utilization can be improved by reducing the BER. For this purpose, efficient multipath and Doppler correction algorithms, as well as efficient error correction mechanisms should be investigated and executed, even in ultra-low-power resource-constrained modems. On the other hand, current acoustic networks are very bandwidth constrained. Thus, in order to increase the total available bandwidth, basic research on piezoelectric transducers should be conducted to obtain high bandwidth and more affordable transducers. Additionally, studies of modulations with higher spectral efficiency using low-power modems should be conducted.

Last, a number of recent and ongoing studies are moving towards platforms to facilitate UWSN experiments [59–61]. Lower layers are implemented using existing commercial modems that cannot be re-configured out of the pre-defined modem settings. These platforms can be remotely reconfigured to perform high-level protocol layer evaluation. Further deployments, with an open access philosophy combined with an easy reconfiguration framework, would lead to faster development of UWSNs.

## 3. Medium Access Control Layer

MAC protocols manage access to the communication medium. Without proper management of the transmission medium, collisions of unrequested communications may degrade overall network performance. Hence, the basic objective of MAC protocols is to avoid collisions. However, they also have to deal with other factors, such as energy efficiency, scalability and latency.

A collision occurs when two or more data frames arrive at the intended receiver simultaneously. Traditional MAC protocols try to handle this time uncertainty by, for example, slotting time (time-division multiple access, or TDMA) or sensing the channel prior to transmission (carrier sense multiple access, or CSMA). However, because of the long propagation delays of underwater transmission, these networks also suffer from space uncertainty, and it is necessary to take into account the locations of the receivers and their possible interferers. This problem is commonly known in the literature as space-time or spatio-temporal uncertainty [62].

Another problem that comes along with long propagation delays is spatial unfairness [62]. Because the packet reception time depends on the distance to the transmitter, the channel becomes free first at the transmitter and later on at the receiver. Hence, nodes closer to the transmitter are able to gain access to the channel before nodes closer to the receiver.

Nevertheless, despite these problems, the two classical schemes in which MAC protocols can be subdivided, *i.e*., contention-free and contention-based schemes, are still valid and used.

**Figure 4.**
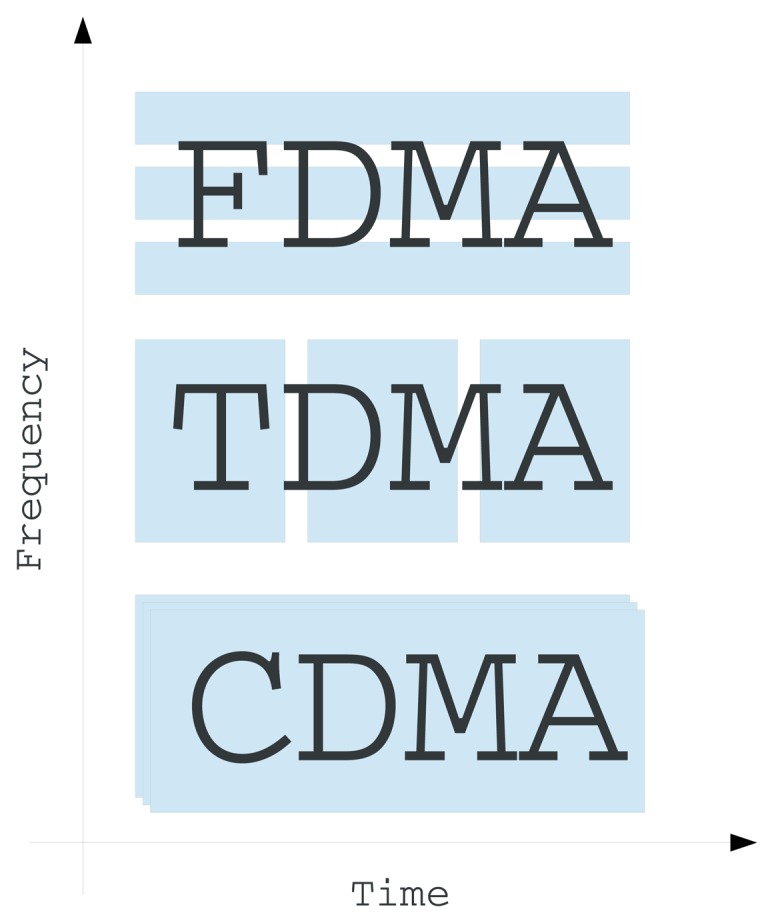
Contention-free communication can be accomplished by assigning time, frequency or unique codes.

Contention-free schemes assign different frequency bands, time slots or codes to different users of the communication medium. Under this scheme, nodes do not compete with each other in order to obtain access to the channel. The three basic types of this scheme, *i.e*., TDMA, frequency-division multiple access (FDMA) and code-division multiple access (CDMA), are illustrated in Figure 4.

Contention-based MAC protocols, on the other hand, avoid the pre-allocation of resources by allowing nodes to compete with each other and obtain medium access on demand. This group of protocols usually relies on random access to distribute transmissions and normally also includes some recovery mechanisms in case a collision occurs.

**Figure 5.**
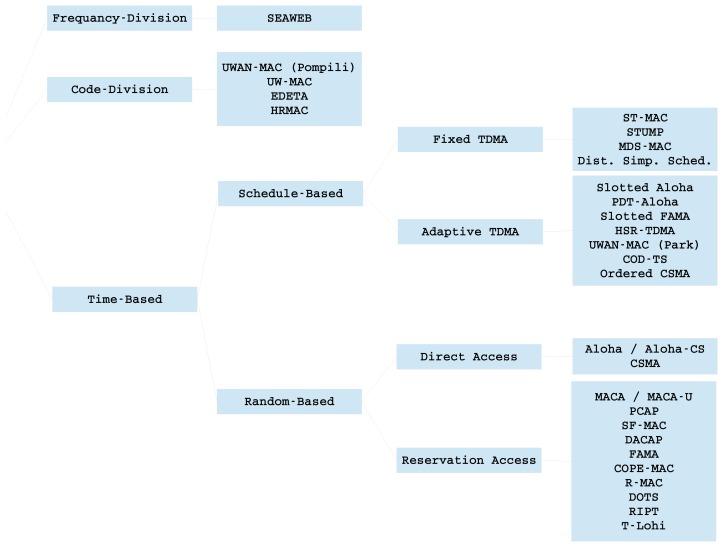
Classification of medium access control (MAC) protocols.

This classification, although widely accepted, is not entirely accurate, because there exist different protocols that share characteristics of both schemes. In Figure 5, we propose a representative classification. The presented protocols are further explained in the remainder of this section, and their main properties are compared in Table 3. This table specifies the type of MAC protocol (time-based, FDMA-based or CDMA-based), whether the protocol uses a random access scheme (CDMA scheme, TDMA scheme or a combination), if the nodes are organized in clusters, whether the MAC protocol also performs routing, if the MAC protocol requires a handshaking process and, finally, if time synchronization is needed or knowledge of the propagation time is required.

### 3.1. Frequency-Division Multiple Access

FDMA is a contention-free medium access scheme that divides the available bandwidth into different frequency bands, allowing different nodes to transmit and receive at the same time while avoiding collisions. However, these frequency bands have to be carefully assigned and used, because collisions may occur inside the same band, as well. For example, if each node is assigned a receiving frequency, when two or more nodes try to reach the same destination at the same time, a collision will occur.

This scheme was used in the early phases of the Seaweb project [63]. Three clusters were deployed, and within each cluster, TDMA was used; FDMA was chosen for inter-cluster communications. However, an inefficient use of bandwidth was reported, and the protocol was also vulnerable to fading and multipath. Since then, FDMA has been considered to be unsuitable for underwater acoustic networks [4,89].

### 3.2. Code-Division Multiple Access

The basic principle of the CDMA scheme is the use of binary codes to modulate the signal using a spread-spectrum technique. When different nodes transmit using different codes with low cross-correlation, their data can be received simultaneously in the same frequency band by the other nodes in the network without collision. The advantage of this scheme over FDMA is that it does not suffer from selective fading, because it uses the entire frequency band. Its advantage over TDMA is that the transmission medium can be accessed at the same time by all users. However, low cross-correlation implies long codes, which greatly reduce the available data rate [37].

It is shown in [37] how the available data rate per user (*V_tx_*) depends on the code length (*L*) and the actual modem data rate ( $V_{tx}^{\prime }$):

(9) $$V_{tx}=\frac{V_{tx}^{\prime }}{L}$$

It is also shown how code length (*L*) depends only on the number of different codes (*N*), as shown in expression Equation (10):

(10) $$L=4^{\log_{2}N}-1$$

Using expression Equations (9) and (10), the effective transmission speed (*V_tx_*) of different users of a network can be calculated. For example, given a network of only eight nodes equipped with an acoustic modem capable of transmitting at 1,000 bps, the effective transmission speed of each node would roughly be 16 bps.

Nonetheless, different authors use the properties of CDMA to their advantage. Pompili *et al.* proposed a combination of CDMA and aloha in [64]. A node willing to transmit dynamically calculates the transmission power and spreading code length in order to efficiently communicate with its intended receiver. This spreading code is then sent using aloha without any sort of coding. Immediately after that, the data packet is sent using the spreading code.

CDMA codes can be used for intra-cluster communication in a clustered network. In [65], the authors propose a cluster-based network in which each cluster has its own CDMA spreading code assigned. Communication inside each cluster is executed by exchanging request-to-send (RTS) and clear-to-send (CTS) packets, and cluster heads communicate with the sink node using TDMA.

Another cluster-based approach is presented in [66], in which, similar to the previous work, each cluster uses its own CDMA spreading code for intra-cluster communication. Transmissions inside the cluster are scheduled using TDMA. Instead of directly communicating to the sink node, cluster heads arrange themselves in a tree structure in order to send their collected data to the sink.

Following this cluster-based proposal, the hybrid reservation-based MAC protocol (HRMAC) [67] utilizes an adaptive TDMA along with CDMA spreading codes. Each node with data to transmit sends a *notice* packet with sender and destination IDs and data size to the cluster head. With this information, the cluster head computes a sending schedule and populates it. Upon receiving the transmission schedule, nodes send their data packets in their assigned slots. Afterwards, the receivers of the data packets send a *reply* message back to the sender with the amount of data received. The CDMA codes are used for the *notice* and *reply* packets, because they are not scheduled and can collide.

### 3.3. Time-Based Schemes

Protocols based on this scheme use the complete bandwidth for a certain amount of time, so multiple transmissions have to be distributed in time by scheduling them, reserving the channel time prior to transmission or directly sending the data packet. Specifically, there are mainly two different strategies to avoid collisions:

1. *Scheduled-based*: a time interval or frame is divided between all nodes. With this strategy, two techniques are normally used:
  (a) *Fixed TDMA*: each node is assigned a time period where it is able to transmit.
  (b) *Adaptive TDMA*: time periods are assigned on demand, either by dynamically assigning the slots by some coordinator or by allowing the nodes to contend for the slots.
2. *Random-based*: selections of the transmission start and end times are arbitrary, and nodes directly compete for channel acquisition. This group of protocols can also be sub-divided into:
  (a) *Direct*. Protocols under this group send data directly without performing any channel reservation.
  (b) *Reservation*. Prior to the transmission of the actual data packet, nodes reserve the channel using control packets.

#### 3.3.1. Scheduled-Based Schemes

In the schedule-based protocols, each node is assigned a time period in which it is able to transmit. This technique requires synchronization between all nodes, which can be done using a synchronization algorithm [90]. In addition, in order to guarantee a contention-free communication, it might be necessary to include guard times. The duration of these guard times depends on the maximum propagation delay and the synchronization accuracy, which degrade the network performance.

There are basically two types of schedule-based protocols, *fixed TDMA* and *adaptive TDMA*. In fixed TDMA, each of these time periods is assigned to a node, and the node is only able to transmit during this time.

Given the large propagation delays in the underwater medium, it is possible that packets from two different nodes arrive successfully, even if the packets were transmitted at the same time [62]. Based on this, different approaches have been proposed that try to schedule the TDMA-based transmissions in such a way that they can overlap without conflicting at the intended receivers.

In [68], the authors propose spatial-temporal MAC (ST-MAC), which formulates the TDMA-based scheduling problem as a vertex-coloring problem. The algorithm constructs a spatial-temporal conflict graph describing the conflict delays among transmission links. Afterwards, an optimal solution is proposed based on a mixed integer linear programming model, and a new heuristic approach is proposed to solve the vertex-coloring problem.

The staggered TDMA underwater MAC protocol (STUMP) is a similar approach introduced in [91]. A set of TDMA scheduling constraints is derived, and the authors propose centralized and distributed algorithms in order to solve the scheduling problem. They first determine the order of transmissions between the conflicting nodes. Once the order is fixed, the scheduling constraints become a system of difference equations, which is solved using the Bellman–Ford algorithm. In a subsequent work [69], the authors further improve their proposal by adding routing capabilities.

A recent alternative is multi-dimensional scaling MAC (MDS-MAC), proposed in [70]. It integrates time-synchronization, localization and communication scheduling for small underwater clusters. The operation of the protocol is divided into coordination and communication phases, which are repeated periodically. During the coordination phase, nodes perform range measurements in order to calculate the propagation delay between them and to achieve relative localization and time synchronization. At the beginning of the communication phase, the cluster head broadcasts the communication schedule and routing information. Afterwards, during the remainder of this communication period, all nodes within the network follow this schedule.

A clustering scheduling approach is described in [71]. Spatial-temporal communication scheduling is performed within clusters by the cluster head. The cluster heads forward the length of the complete schedule to a central scheduler, which assigns time to the different clusters. By allowing the cluster heads to schedule within the cluster, the central scheduler does not need to know the positions of all nodes within the network to guarantee a collision-free schedule. This reduces the otherwise very significant overhead of spatial-temporal communication scheduling.

Ordered CSMA [78] schedules transmissions through ordering. Every node in the transmission chain waits until it has detected the carrier of the preceding node in the schedule. After detecting the carrier, the node is allowed to transmit its data. By ordering the transmissions in such a way, collision-free transmissions are guaranteed.

On the other hand, adaptive TDMA protocols allow nodes to adaptively assign time periods on demand. This assignment can be done through contention and handshaking processes or by learning the transmission schedules of the neighboring nodes.

In slotted aloha, as in pure aloha, nodes contend for the channel [72]. However, in slotted aloha, the transmission is deferred to the beginning of each time slot. Hence, each node is obliged to schedule the beginning of its transmissions at the beginning of each time slot. Nevertheless, because slots are not assigned as in pure TDMA, collisions may also occur if different nodes select the same slot to transmit. However, given the space uncertainty of the underwater acoustic medium, the performance of this protocol is degraded to that of pure aloha. In [73], the authors try to cope with this problem by adding extra guard time in the time slots, achieving 17%–100% better throughput results than the original slotted aloha in an underwater medium.

The original floor acquisition multiple access (FAMA), which is introduced in Section 3.3.2., requires long RTS and CTS packets in order to guarantee that the data packets will be transmitted collision-free [83]. However, in the underwater acoustic channel, where transmissions are expensive, excessively large control packets might be too energy expensive. In order to reduce these high energy costs, slotted FAMA uses time slots, in the same way as slotted aloha, to reduce the control packet size [74]. The slot length is equal to the maximum propagation delay plus the transmission time of a CTS packet, which assures that only control packets may collide and that the transmission of data packets is collision-free.

Another approach is the one proposed in [75], in which TDMA is used, but nodes are able to adaptively identify who can transmit at the same time without causing collision. In order to do so, nodes calculate a list of neighbors and share it by piggybacking it to their outgoing packets. Upon reception of a new list of neighbors, each node updates its connectivity matrix, and based on this, the node then decides whether or not it is able to safely transmit in the next slot.

A different proposal for low-duty-cycle underwater communication networks is given in [76]. This protocol sets up an adaptive TDMA schedule. Nodes first exchange SYNCpackets within their transmission periods and learn their neighbors. Consequently, a node knows when it should wake up to hear a transmission and when there are no transmissions, so that it can remain in sleep mode.

Cluster-based on-demand time sharing (COD-TS) [77] proposes a different solution. Nodes are organized into clusters, and the cluster heads are the nodes in charge of assigning the slots for the next communication round. At the beginning of each round, the cluster head populates the schedule, and each node sends its request to transmit at the end of the communication round. In addition, cluster heads communicate among themselves in order to avoid collisions with neighboring clusters.

#### 3.3.2. Random-Based Schemes

This set of protocols avoids the pre-allocation of resources by allowing nodes to compete with each other and obtain medium access on demand. These protocols usually rely on random access to distribute transmissions. They also normally include some recovery mechanisms in case a collision occurs. Protocols under this category can be subdivided into direct access, handshake access and contention access.

##### Direct Access

Protocols under this category do not perform any kind of handshake for channel reservation. However, they can perform carrier sensing prior to transmission in order to avoid disrupting ongoing transmissions, deferring it until the channel is free.

Aloha [72] is the simplest method to access the medium. It simply sends a packet whenever there is data to send, without performing any type of channel assessment or retransmission. In its variant, called aloha with carrier sensing (aloha-CS), prior to transmission, each node performs a clear channel assessment (CCA) in order to avoid disrupting any ongoing transmission. If the channel is sensed to be free, the sending node transmits its packet. However, if an ongoing transmission is sensed, the node waits until the channel is free and then sends the packet.

Different studies have been conducted in order to understand the performance of aloha in an underwater medium. In [92], the authors develop an analytic model to study the performance of contention-based protocols by modeling different versions of aloha in an array network. Although the authors make several simplifications, different conclusions are drawn. Arrays longer than five hops are probably not going to perform well when using the simple aloha protocol. However, using p-persistent aloha without dropping packets increases the network throughput at the cost of increasing the delay.

Another study based on the aloha protocol is presented in [93], in which two different variants of aloha are proposed. Aloha with collision avoidance (aloha-CA) tries to avoid collisions by overhearing the transmitted packets and knowing the propagation delays between all node pairs. The other proposed modification is aloha with advanced notification (aloha-AN), which consists of sending a short data packet prior to the actual data transmission with information on the sender and the intended receiver.

CSMA [72] is another well-known protocol under this category. Similar to aloha-CS, this protocol uses carrier sensing. However, unlike aloha-CS, after waiting for the channel to be free, a node does not immediately send its packet. Instead, it performs random back-offs to mitigate the probability of collisions. This variant is called non-persistent CSMA. In another variant, called p-persistent CSMA, a node transmits with a probability of p when the channel is sensed to be free. Note that 1-persistent CSMA is equivalent to aloha-CS.

##### Reservation Access

This technique consists of reserving the channel prior to the transmission of the actual data packet. In order to do so, usually, short control packets are sent before transmission. By reserving the channel, the frequency of the collisions of data packets is minimized, and the additional traffic introduced by the control packets is compensated for.

In the handshake-based alternative, whenever a transmitter wants to send a data packet, it first sends a control packet informing the other nodes that it has data to send. Upon reception of this control packet by the intended receiver, it replies if the channel is not being used. After receiving this reply packet, the transmitter can start the transmission of the data packet.

However, this reservation mechanism cannot guarantee collision freeness, because of the well-known hidden and exposed node problems. Many authors have proposed different solutions to cope with this problem.

Multiple access collision avoidance (MACA) [94] is the first approach of a handshake algorithm proposed to reserve the channel. The sender first sends an RTS control packet in order to start the channel reservation. This packet contains the length of the data packet, so that other nodes know how long they should wait until they can initiate their own transmissions. Afterwards, the receiver replies with a CTS packet. When the transmitter receives the CTS, it starts the transmission of the actual data packet. However, this algorithm does not entirely solve the exposed terminal problem, especially in channels with long propagation delays, such as the underwater channel. MACA for underwater (MACA-U) [79] adapts MACA to the long propagation delays of the underwater medium by modifying some of the state transition rules of the original protocol.

Another protocol to solve the handshaking problems in the underwater medium is the propagation-delay-tolerant collision avoidance protocol (PCAP) [80]. This protocol splits the transmission of a CTS packet, so that it arrives at the transmitter after twice the maximum propagation delay. While waiting for the CTS packet, the transmitter and its neighbors can perform different actions, such as transmitting data packets or starting the handshaking process for another transmission.

Spatially fair MAC (SF-MAC) [81] also tries to avoid collisions by deferring the CTS packet transmission for a predefined amount of time. During this time, the receiver analyzes all RTS packets that are sent to it and determines, based on an estimate, which node was the first to send the RTS packet and the node to which the CTS packet should be addressed.

Another random access with reservation protocol is the distance-aware collision avoidance protocol (DACAP) proposed in [82]. This protocol tries to avoid data and RTS packet collisions by deferring the data transmission for *t* seconds after sending the RTS. This waiting time has to be chosen based on a trade-off between throughput and collision probability. Moreover, it also introduces a short warning packet sent by the receiver if it overhears an RTS after sending a CTS. Another approach is given by the original FAMA protocol [83], which completely prevents data packet collisions, provided that the RTS and CTS frames are sufficiently long. The length of an RTS packet should be greater than the maximum channel propagation delay, and the length of the CTS packet has to be greater than the length of an RTS plus one maximum round-trip time. In order to introduce some energy savings, Molins *et al.* propose in [74] the slotted FAMA protocol described in Section 3.3.1..

By reserving different transmissions in one multiple reservation packet broadcast to all neighbors and trying to arrange data transmission with several nodes, contention-based parallel reservation MAC (COPE-MAC) [84] improves channel utilization. Moreover, neighboring nodes can, by overhearing, learn about future scheduled transmissions and adapt their own channel utilization to avoid collisions.

The reservation-based MAC (R-MAC) protocol is proposed in [85] and is designed for long-term monitoring applications. Nodes alternate between sleep and listen modes periodically and randomly select their schedule. The protocol requires all nodes to know the propagation delay to all of their neighbors and their listen and sleep periods. Afterwards, the protocol reserves the channel in an RTS/CTS fashion, but gives higher priority to the CTS packets.

Another protocol that exploits spatio-temporal uncertainty is delay-aware opportunistic transmission scheduling (DOTS) [86], which exploits temporal and spatial reuse by learning the propagation delay to neighboring nodes and their scheduled transmissions. In order to achieve this, nodes must be synchronized and continually overhear the channel. The protocol is based on a MACA-like random access protocol with RTS and CTS packets. By promiscuously overhearing, a node using DOTS can locally calculate the transmission and reception schedules of its neighbors and schedule on its own to avoid collisions.

Receiver-initiated packet train (RIPT) [87] is different from the previous protocols, as it employs a receiver-initiated four-way handshake mechanism. Instead of the sender, the node that initiates the handshaking process is the receiver, which informs its neighbors that it is available to receive. After that, the neighboring nodes inform the receiver about the size of their transmissions, and with that information and the previously known propagation delay, the receiver can calculate and broadcast a transmission order. Finally, senders follow this transmission order, and the data arrives at the receiver in a sequence of packets.

Tone-Lohi (T-Lohi) [88] implements this technique. It automatically adapts the contention time to the number of contending nodes. The nodes send a short packet, called tone prior, to the actual data packet to count the number of terminals contending for the channel. If a node does not receive any other tones, it starts the transmission. If it receives more tones, it adapts its back-off time, depending on the number of tones received.

### 3.4. Security at the MAC Layer

At this layer, jamming attacks can also be performed. In order to perform this type of attack at the MAC layer, the attacker needs to know some information about the protocol being used. The jammer uses well-formed, legitimate control or data packets instead of noise in order to occupy the channel. Again, as for the physical layer, different solutions have been purposed for WSN [57].

This attacks along with Sybil attacks and packet spoofing can be mitigated by using authentication. In this manner, nodes would know if a packet is sent by a legitimate node or it has been inserted in the network by a malicious node. How this authentication should be performed and how the encryption keys are disseminated through the network has been addressed in [95] for higher layers of the network stack hence, further research should be conducted.

### 3.5. Conclusions and Future Challenges

There are a handful of MAC protocols proposed for the UWSN. However, each protocol tries to solve different issues or gives more importance to one characteristic over another. There are many different applications with many different parameters and requirements, and choosing one MAC protocol over another can be a difficult task. Although there has been some work done on the performance analysis of different MAC protocols for a given application [14], further research should include guidelines to choose between the different MAC alternatives depending on the target application constraints.

In addition, MAC protocols that require synchronization or localization do not usually take into account the energy consumption of these two services. MDS-MAC [70] is a recently proposed protocol that integrates these two services into its specifications and takes into account the extra energy consumption. Newly proposed protocols that make use of these services should integrate them into their behavior for a complete evaluation.

Finally, future challenges in the development of MAC protocols for acoustic UWSNs shall include the use of new modem features such as wake-up systems, which can greatly reduce power consumption. Only one protocol, T-Lohi [88], makes use of this new characteristic. The applicability of the wake-up system to the existing MAC protocols needs to be evaluated, and new protocols utilizing this technique shall be proposed.

## 4. Routing Layer

Routing is an important aspect in any multi-hop network. The routing layer is in charge of selecting the most convenient paths in order to deliver data towards the destination. In some underwater network applications such as rescue missions, short network deployment times are required. Moreover these applications do not allow for previous deployment planning. In such scenarios, routing protocols must be able to determine the best routes to the destination without any pre-existing network knowledge.

The currently existing routing protocols for terrestrial networks can be classified into two major categories: proactive and reactive. On the one hand, proactive routing protocols have a large signaling overhead every time there is a topology change, which occurs continuously underwater with frequent node movements. On the other hand, reactive routing protocols, which are designed for dynamic scenarios, have large delays and are heavily aggravated underwater.

**Figure 6.**
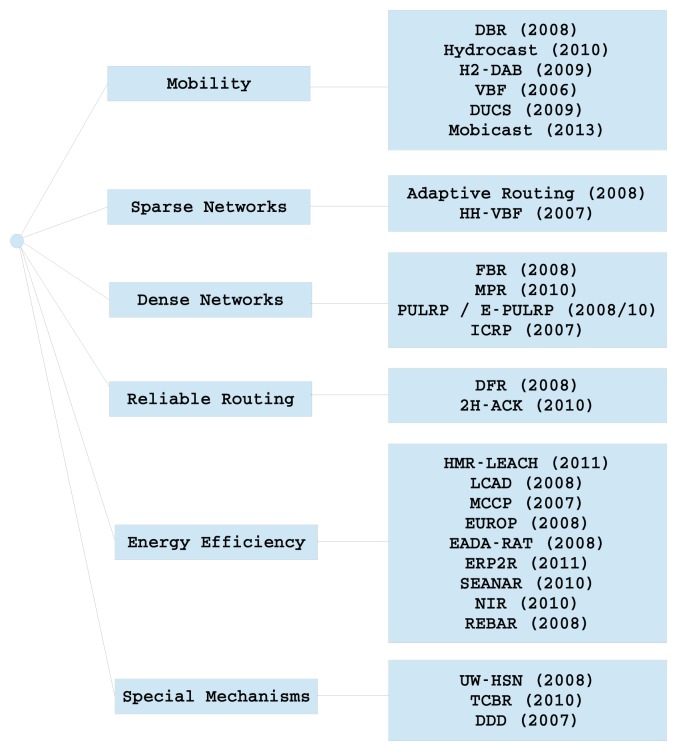
Classification of routing protocols.

In this section, the main routing protocols for UWSNs are discussed and shown in Figure 6. Efficient routing is a key element in these types of networks. Hence, routing protocols need to take into account their special features and the requisites of new application fields for UWSNs. The presented protocols can be classified in many ways according to different criteria weighting. The classification that has been followed meets the criteria indicated in each sub-section, although many protocols could belong to several classes.

### 4.1. Mobility

In this sub-section, routing protocols that support mobility are discussed. There are many underwater networks applications where mobility is required at least in certain nodes; hence, different authors have tried to cope with this problem.

In depth-based routing (DBR)for underwater sensor networks [96], each sensor calculates the forwarding action, taking into account its depth and the depth of the previous node. In this greedy algorithm, when a node has data to send, it broadcasts the message. Depth calculation and comparison are executed by neighbor nodes. In this line, nodes with shallower depths than the sender accept messages while dropping the other ones. The main disadvantage of DBR is that all nodes need a depth sensor, increasing the consumption and cost. The required broadcasting is another disadvantage. Finally, it is important to show the significant difference in performance when the node density changes.

A similar approach is the one given by HydroCast [97], which is intended as an alternative to geographic routing, by using anycast routing and the pressure level with the goal of forwarding the data messages up to the surface. In this manner, is not necessary to implement an expensive distributed localization mechanism. HydroCast takes routing decisions after a depth information comparison, forwarding packets in a greedy manner towards a node with minor pressure using its neighbors. HydroCast assures that each local maximum node maintains a recovery path towards a neighbor with shallower depth. In this manner, a data packet can be routed out of the void region being switched back to the greedy mode. Simulation results show that HydroCast provides elevated delivery ratios with reduced delays.

Instead of using depth sensors, in a hop-by-hop dynamic-addressing-based (H2-DAB) protocol [98], data is sent from nodes towards the water surface in a greedy fashion, assuming multiple buoys on the surface, which collect data from nodes deployed at different depths. Each floating node is assigned a dynamic ‘hop_id’, and anchored nodes have only one static ‘hop_id’. On the other hand, surface and floating nodes have two addresses. On the water surface are located the sinks which must send ‘hello’ messages with the maximum hop count to other nodes to allow them to achieve the ‘hop_ids’. The ‘hop_id’ of the floating nodes can change only after the reception of a ‘hello’ message. The node forwards the ‘hello’ message and the changed ‘hop_id’, after the ‘hop_id’ upgrade, to its neighbor and decreases the value of the hop-count. When the ‘hello’ message is received by an anchored node or the hop count is zero, the procedure is finalized. Evaluation results conclude that the density of the nodes does not influence the delivery ratio; the main drawback of this protocol is the high delay.

A well-known location-based routing protocol for USNs is the vector-based forwarding protocol (VBF) [99]. VBF connects a routing pipe between the sender and receiver. All data transmission must use this pipe, with each packet containing the position of the sender, forwarder, receiver, and a ‘range’ field used for mobility implementation. Results show that VBF is intended for a dense USN, reducing the size of the pipe for network traffic. On the other hand, for VBF, disadvantages are possible in that node density influences the pipe efficiency. It is possible that in sparse networks, the pipe does not have sufficient nodes to forward messages.

A clustering approach is described by the distributed underwater clustering scheme (DUCS) [100], which is intended for scenarios with random mobility that do not use geographical information. DUCS organizes nodes into clusters, and each node is one hop away from a cluster head. The head role is alternated. The protocol operation is divided into two phases: configuration and communication. The cluster head receives data from the cluster nodes executing aggregation. Finally the heads send the data to the sink by means of multi-hop. An evaluation has concluded that DUCS has an increased throughput and lower overhead than low-energy adaptive clustering hierarchy (LEACH).

Finally, the so-called mobicast routing protocol [101] is intended to improve throughput and efficiency. This algorithm has two steps. Autonomous underwater vehicles gather messages from nodes within a 3D zone of reference in the first step. After that, they wake up the sensor nodes in the next 3D zone, avoiding topology holes. In mobicast, disadvantages are possible, considering that the delivery rate and energy efficiency decrease with high water flow velocity.

### 4.2. Sparse Networks

In this sub-section, routing protocols mainly intended for sparse networks are discussed. An increasing number of applications for this type of network are emerging because deployment costs in underwater networks are higher than those in RF networks.

Routing in the adaptive routing protocol [102] is based on the type of packet and requirements, adaptively obtaining high delivery ratio, efficiency, and reduced delay. The application scenarios are 3D sparse USNs divided into layers. USN nodes can move in the 2D horizontal plane and can be deployed at different depths. Nodes use ‘hello’ and data packets executing a geographic routing scheme. Packet priority is computed taking into account the message emergency level, age, node energy, and density.

On the other hand, the hop-by-hop vector-based forwarding (HH-VBF) protocol [103] uses the concept of a routing pipe, similar to VBF. The main difference is that HH-VBF creates a pipe for each forwarder. This algorithm enhances the main problems of the VBF protocol. HH-VBF overcomes the previously stated problem found in sparse network forwarding nodes. Simulation results show that HH-VBF enhances the original VBF in terms of delivery rate and by being more energy efficient. On the other hand, the main VBF disadvantages are that node density greatly affects the efficiency of creating a pipe from source to destination. Additionally, the choice of thresholds can affect the routing performance significantly.

### 4.3. Dense Networks

In this sub-section, the routing protocols mainly dealing with dense networks are discussed. Such networks are characterized by high node density in the geographical area of the network deployment.

The main goal of focused beam routing (FBR) [104] is to reduce flooding. Every node knows its location and the end destination location. This protocol defines the paths during the traversing of a data packet in a dynamic manner, calculating the next hop at each step. On the other hand, FBR presents some problems, e.g., the possibility that no node will lie within the appropriate area because of nodes becoming sparse owing to water movements. Additionally, when a node is unable to find the next relay node, it needs to send the RTS again (by broadcast), thus increasing the overhead and affecting packet deliveries in sparse zones.

Another protocol that dynamically calculates routes is multipath routing (MPR) [105], which constructs a route from sender to receiver using several multi-sub-paths, which are sub-paths from the sender to the two-hop neighbors. In this manner, relay nodes check the transmission schedule without delay to verify if a collision has occurred. The forwarder node must defer transmission in the case of collision. Results shows that MPR outperforms other approaches, but its main disadvantage is that at low velocity, it has a higher overhead value.

A different approach is the one followed by the path-unaware layered routing protocol (PULRP) [106], which is intended for dense 3D USNs. The first step of this algorithm is dedicated to a layering task, where spheres are structured around a sink. In the second step, the selection of the intermediate nodes and normal communication is performed. The main properties of PULRP are that it does not require fixed routing tables, synchronization, or localization. Its main advantages are its good delays and message delivery rate. However, its main disadvantage is that the radii of the spheres have a significant impact on message delivery.

Further improvements were made to this protocol in order to achieve improved energy efficiency. Energy optimized path unaware layered routing protocol (E-PULRP) [107] improves the previous PULRP protocol from a networking energy-balance point of view. Energy balance is performed by selecting the widths of different layers and calculating the minimum consumption and the probability of successful transmissions. The goal of this algorithm is to face the mobility problem without requiring synchronization or localization. The main disadvantages of this approach are that, depending on the scenario, the relation between consumption and throughput varies and impacts the delays.

Finally, the information-carrying-based routing protocol (ICRP) [108] is a reactive protocol intended for scalable routing and reduced consumption. The routing task is performed by a small number of nodes. The sender initiates the path establishment. The sender must broadcast the message in case there is no established path, carrying the path discovery packet. The nodes that receive this message must broadcast it and save the reverse route. When the final receiver node gets this message, it is possible to obtain the complete reverse route. All routes have timeouts, with it being necessary for a route to be discovered by the defined timeout.

ICRP evaluation highlights several performance drawbacks. Because decisions are based on saved information, ICRP is not appropriate for underwater networks where nodes are in continuous movement. The other relevant drawback is due to the necessity to broadcast packets if one node does not have a route to reach a destination, which increases power consumption.

### 4.4. Reliable Routing

In this sub-section, the major USN routing protocols focusing on reliability proposed to date are reviewed. Reliability in routing for UWSNs is usually achieved by maintaining different copies of the packets being sent through different paths towards the destination. A greedy and straightforward technique to implement this is packet flooding.

One protocol that implements the flooding technique is directional-flooding-based routing (DFR) [109]. This protocol has been proposed in order to improve reliability. DFR avoids flooding by means of performing the routing tasks for one message with only a reduced number of nodes. This protocol assumes that all nodes have location information, and the forwarding nodes are selected as a function of the signal strength. Results show that DFR throughput is a function of the set of forwarding nodes selected for the link.

Instead of flooding, in two-hop acknowledgments (2H-ACK) [110], two instances of one message are maintained in the USN without an additional load, increasing network reliability.

### 4.5. Energy Efficiency

In this sub-section, routing protocols that focus on energy efficiency are presented. Energy is a fundamental aspect in the type of network deployed; hence, it is usually the main topic in research literature in addition to throughput and delay.

One widespread technique in radio frequency sensor networks which can be applied underwater is clustering. Hierarchical multi-path routing low-energy adaptive clustering hierarchy (HMR-LEACH) [111] is an enhancement of classic LEACH [112]. In HMR-LEACH, cluster heads must transfer data to the sink using multi-hop, and route selection is performed by assigning probabilities. HMR-LEACH is an appropriate algorithm for large-scale underwater networks because it prevents long-distance transmissions. Experiments show that this approach improves energy efficiency when compared with LEACH and LEACH-M [113].

Another approach that utilizes clusters is the location-based clustering algorithm for data gathering (LCAD) [114]. Its main goal is to improve the behavior of sensor nodes near the sink which rapidly consume their battery power. It is a protocol for 3D UWSNs where sensor nodes are deployed at fixed relative depths, being organized in clusters. An algorithm which takes into account the node location is used for cluster head choice. In LCAD, each cluster will have multiple cluster heads. In order to implement intra-cluster communication, nodes transmit through the horizontal links.

The communication protocol during configuration performs the cluster-head designation. Afterwards, LCAD performs intra-cluster communication (nodes send messages to their head) and inter-cluster communication (heads send collected data to the sink being aided by autonomous underwater vehicles). LCAD behavior depends on the USN structure. Specifically relevant for the performance is the location of cluster heads. In USN, nodes can change location within the grid frequently because of underwater node movement, impairing performance.

Continuing with the clustering approach, the main goal of the minimum-cost clustering protocol (MCCP) [115] is to enlarge the underwater network life, taking into account the non-uniform node distribution due to continuous movement of ocean currents. MCCP defines a cluster structure, being the clusters established as a function of the energy required by the nodes to transmit packets to the head, the position of the head and the sink, and the energy of the nodes. The heads must calculate a TDMA schedule that must be sent to the nodes inside the cluster.

This protocol generates more heads near the sink, avoiding the creation of hot spots. This algorithm does not support multi-hop routing, and the period for the cluster definition is normally very large. This period will influence consumption in target environments where nodes are in continuous movement, moving between various clusters in this interval.

The EUROP protocol [116] focuses on energy efficiency and response waiting time, mainly reducing broadcast packets. Each node is equipped with a pressure sensor in order to calculate its depth position, eliminating the necessity for ‘hello’ packets. The base station located on the water surface can receive information only from shallow water nodes. Detecting the pressure value, sensor nodes determine their corresponding layer and use defined messages to communicate through the acoustic channel, selecting the next hop by applying the rule from deep to shallow nodes. EUROP is not a complex protocol, but the cost per node will increase because of the necessary devices that upon addition will increase consumption.

By minimizing the number of transmissions and moving the aggregation point, energy-aware data aggregation via reconfiguration of the aggregation tree (EADA-RAT) [117] improves the consumption and delays. This protocol reconfigures the tree by means of a dynamic pruning procedure to obtain temporal routes.

Another approach is the one followed by the energy-efficient routing protocol based on physical distance and residual energy (ERP2R) [118] which tries to minimize consumption by improving the delays while taking into account residual energy and distance in order to minimize unnecessary retransmissions.

A simpler protocol is the so-called SEANAR [119]. For path calculation, SEANAR adopts a more efficient technique than VBF and greedy forwarding, assigning weights to sensors that have greater connectivity with the base station.

Similarly, neighbor information routing (NIR) [120] selects the next hop by applying a greedy approach which provides a greater transmission rate to nodes with a high number of neighbors. With the objective to obtain reduced consumption, this algorithm follows the simple greedy forwarding technique. The main disadvantage is that NIR is only intended for 2D underwater scenarios; however, this approach achieves high efficiency, which is its main design goal.

The reliable and energy balanced routing algorithm (REBAR) [121] utilizes a limited broadcast by implementing an adaptive algorithm in order to adjust the propagation range, taking into account energy efficiency. This protocol broadcasts in a specific domain, applying geographic information, instead of in an entire network broadcast, in order to minimize battery usage of the nodes around the sink. Results show that REBAR performance is better than VBF-based protocols in terms of USN lifetime and message delivery. Nevertheless, without mobility, the nodes around the base station deplete the batteries rapidly, impeding the correct functioning of the network.

### 4.6. Special Mechanisms

In this sub-section, the routing protocols which use special physical mechanisms or devices in order to operate in an efficient manner are discussed. These special physical mechanisms can include, for example, mechanical modules that regulate sensor depth [122,123], low-power wake-up systems [124], or autonomous underwater vehicles (AUVs) which have to harvest data from actual sensor nodes [124].

The underwater wireless hybrid sensor network (UW-HSN) [122] protocol is a mix of acoustic and radio communications. UW-HSN uses radio communication for continuous traffic and acoustic communication for reduced data messages. In this manner, all nodes are equipped with radio and acoustic interfaces, using the acoustic interface for underwater communication and the radio interface if nodes are on the surface communicating with the sink. All nodes are equipped with a mechanical module in order to help the node to swim to the surface and dive back down to different underwater levels. Simulation results show that this protocol presents elevated throughput with reduced delays, but no results have been reported about the consumption and cost, which are key factors in this case because of the necessity of special hardware and devices.

In temporary cluster-based routing (TCBR) [123], only special designated nodes need to include a mechanism to regulate their depth. This protocol is intended to solve the problem of multi-hop routing where nodes near the sink multiply their consumption. The idea is to deploy multiple sinks, and messages received at any sink are correctly received because they can communicate at an elevated data rate and have small propagation delays using RF communication. In TCBR, normal nodes are in charge of sensing and forwarding data to the nearer courier nodes. In order to promote energy consumption, a reduced set of courier nodes is used, and these can sense as well as receive the data from normal sensor nodes and forward them to a surface base station. The courier nodes dispose of a module that allows them to push the node down into the water at different depths and pull back the node to the surface. Courier nodes broadcast ‘hello’ packets at each defined depth in order to allow the nearest normal node to send the courier node the data. Sensor nodes must save their generated data in a buffer until a courier node appears in the vicinity; thus, it is impossible to provide a response in bounded time with this approach. Moreover, is possible that a node loses packets while awaiting the courier node's presence.

Finally, the delay-tolerant data dolphin (DDD) [124] protocol follows another approach. It is intended to increase energy efficiency for delay-tolerant scenarios. This protocol uses dolphins, which are special mobile nodes that must visit the sensor nodes in order to retrieve their measured data. Using this technique, each sensor node must only send its locally stored data in one hop to the nearest dolphin node. Because it is unnecessary to use multi-hop routing, DDD saves energy in this manner in the sensor nodes. Dolphin nodes transmit a special signal, and in this manner, the acoustic modem must implement a low-power transceiver to detect the closeness of a dolphin node. Dolphin nodes will provide collected messages when approaching a sink on the water surface. When judging this protocol, the number of dolphin nodes is a key factor. It is necessary to have an equilibrated number of dolphin nodes in order to visit all sensor nodes frequently to prevent data loss, taking into account that an excessive number of dolphins will significantly increase the cost.

### 4.7. Security at the Routing Layer

There are several attacks that can be detrimental to routing protocols like sinkhole attacks, Sybil attacks, selective forwarding attacks or wormhole attacks. In [125] a taxonomy of different vulnerabilities, attacks and countermeasures for UWSN is provided.

In a sinkhole attack a malicious node tries to deviate packets towards it. The selective forwarding attack consist on malicious nodes dropping selected messages in order to sabotage routing. Sybil attacks are based on a device taking multiple identities. All this attacks can be mitigated by using authentication. In [126] authors purpose a security suite at the communication middleware level based on the group communication paradigm. The nodes that form a group share a key and use it to encrypt and authenticate messages. To ensure confidentiality messages are encrypted using AES and authenticity is achieved by truncating the real hash function value (SHA-256) to a 4 bytes digest hence, reducing the total overhead to the expenses of reducing security. When a node is believed to be compromised it is forced to leave the group and a new key is then distributed using the S2RP protocol [127]. Furthermore, in a subsequent work [95], the authors apply these same ideas to the routing layer.

In a wormhole attack, a malicious node transmits packets received in one end of the network over a low latency link to another end causing false neighbor relationships, thus affecting routing. Countermeasures against the wormhole attack for UWSN are presented in [128] where every sensor collects its distance to its neighbors and broadcasts it. By doing so, every node is able to construct the local topology and wormholes detected.

### 4.8. Future Challenges

In this section, known open research issues are discussed which must be considered during future work on underwater environments. In the case of failure, routing must be self-configuring. According to the underwater environment, algorithms should provide strict or loose latency bounds for time-critical applications. For delay-tolerant applications, protocols must try to develop mechanisms to handle a loss of connectivity without immediate retransmissions. A handful of security solutions have been purposed for WSN [129]. However security is a relatively new research topic in UWSN that needs to be further explored.

In summary, future work in this area must consider intelligence techniques, QoS, node mobility and security. In applications where nodes are mobile, new routing protocols are needed to handle the frequent topology changes and to ensure reliable delivery.

## 5. Conclusions and Future Trends

This paper presents a comprehensive view of the current state-of-the-art in UWSNs by analyzing the current research status of the physical, MAC and routing layers. The interaction of these layers is essential in order to advance the research and development of UWSNs. Hence, each layer must account for the advances of the others and use them to its advantage.

On the physical layer, further research should be conducted to implement complex algorithms and modulations in low-power, low-cost micro-controllers. These algorithms should try to achieve better bandwidth utilization while reaching further transmission distances in an energy-efficient manner. One line of action can be reducing the bit error rate by developing efficient multipath and Doppler correction algorithms, as well as efficient error correction mechanisms. At the same time, research on applying modulations with high spectral efficiency for UWSN and high bandwidth piezoelectric transducers should be conducted.

In turn, on the MAC layer, protocols should take advantage of hardware advances, like low-power wake-up mechanisms. Current protocols need to be adapted in order to evaluate them and study their performance, since only the T-Lohi protocol implements this new characteristic. In addition, several MAC protocols require localization and synchronization mechanisms, which are usually not taken into account when evaluating their performance. Further studies need to include this services in order to accurately evaluate their performance.

Following that, in the routing layer, new protocols should account for intelligent self-configuring techniques in order to handle frequent topology changes and reliable delivery. Mechanisms to handle connectivity loss should be further studied for delay-tolerant networks. On the other hand, time-critical applications need latency bounds, which are difficult to provide in an underwater medium.

Finally, security is an issue that needs to be tackled at all layers of the protocol stack. Although there are a handful of proposals for WSNs, research on the topic of security for UWSNs is still in its infancy. Preliminary jamming studies have been performed, as well as some promising techniques that are mostly focused at the routing layer.

## Figures and Tables

**Table 1.** Analysis of commercial modems. AT-WU, acoustic-triggered wake-up; FSK, frequency-shift keying; PSK, phase-shift keying.

**Modem**			**Frequency (kHz)**		**Bit Rate (bps)**	**Distance (km)**	**Power**			
**Company**	**Model**	**Initial Version**	**Center**	**Bandwidth**			**TX(W)**	**RX(W)**	**Sleep (mW)**	**AT-WU (mW)**
**Aquatec** [51]	AQUAModem 500	1998	29	4	100	-	-	-	-	-
	AQUAModem 1000	2009	9.75	4.5	2,000	5	20	0.6	1	5
**DSPComm** [53]	AquaComm Marlin	2003	23	14	480	1	1.8	0.252	1.8	25.2
	AquaComm Mako	2003	23	14	240	1	1.8	0.252	1.8	25.2
	AquaComm Orca	2003	23	14	100	3	1.8	0.252	1.8	25.2
**TriTech** [54]	MicronModem	2008	22	4	40	0.5	7.92	0.72		-
**WHOI** [52]	MicroModem (FSK)	2005	25	4	80	2	100	0.23	0.2	-
	MicroModem (PSK)	2005	25	5	5,388	2	100	2.23	0.2	-
**TELEDYNE BENTHOS** [7]	ATM9XX (PSK)	2011	11.5/18.5/24.5	5	2,400	6	20	0.768	16.8	-
	ATM9XX (MFSK)	2011	11.5/18.5/24.5	5	15,360	6	20	0.768	16.8	-
	ATM885	2002	18.5	5	15,360	0.7	84	0.7	-	-
**EvoLogics** [6]	S2CR 48/78	2005	63	30	31,200	1	18	1.1	2.5	5/285
	S2CR 40/80	2005	51	26	27,700	1	40	1.1	2.5	5/285
	S2CR 18/34	2005	26	16	13,900	3.5	35	1.3	2.5	5/285
	S2CR 12/24	2005	18.5	11	9,200	6	15	1.1	2.5	5/285
	S2CR 7/17	2005	12	10	6,900	8	40	1.1	2.5	5/285
**LinkQuest** [55]	UWM1000	2000	35.695	17.85	17,800	0.35	1	0.75	8	8
	UWM2000	2000	35.695	17.85	17,800	1.5	2	0.8	8	8
	UWM2000H	2000	35.695	17.85	17,800	1.5	2	0.8	8	8
	UWM2200	2000	71.4	35.7	35,700	1	6	1	12	8
	UWM3000	2000	10	5	5,000	3	12	0.8	8	8
	UWM3000H	2000	10	5	5,000	3	12	0.8	8	8
	UWM4000	2000	17	8.5	8,500	4	7	0.8	8	8
	UWM10000	2000	10	5	5,000	10	40	0.8	9	8
**Desert Star Systems** [56]	SAM-1	2008	37.5	9	154	1000	32	0.168	-	-

**Table 2.** Comparison of research modems. OFDM, orthogonal frequency-division multiplexing; DSSS, direct-sequence spread-spectrum; OOK, on-off keying.

**Institution**	**Modem**	**Presented**	**Platform**	**Center Frequency (kHz)**	**Range (km)**	**Bit Rate (bps)**	**Modulation**
University of Southern California	USC [30]	2006	ATMEGA128L MCU(Mica2)	18	0.5	600	FSK
University of California, Irvine	UCI [31]	2006	Tmote (MSP430 MCU)	1.5	0.01	48	8-FSK
University of Connecticut	uConn [38]	2007	TMS320C6713 DSP	12.5	(Lab)	6,200	OFDM (QPSK)
Massachusetts Institute of Technology	rModem [35]	2006	TMS320C6713 DSP	12	0.016	550	QPSK
University of California, Santa Barbara	AquaModem [36]	2005	TMS320C6713 DSP	24	0.44	133	DSSS
Kookmin University	Kookmin [29]	2009	ATmega128 MCU	30	0.03	5000	OOK
Massachusetts Institute of Technology	AquaNode [32]	2007	ADBlackfin B533 DSP	30	0.4	300	FSK
University of California, San Diego	UCSD [2]	2010	FPGA				
Northwestern Polytechnical University in China	NPUC [39]	2007	ADSP-TS101 + FPGA	N/A	(Lab)	1000	OFDM (BPSK)
North Carolina State University	NCSU [33]	2008	Atmega 168 MCU	47.5	0.1	31	4-FSK
Gangneung-Wonju National University	GaWoNU [28]	2012	ARMCortex-M3	70	0.07	200	OOK
ITACA Institute	ITACA [48]	2012	C8051F920	85	0.1	1000	Coherent FSK

**Table 3.** Protocol classification and properties. TDMA, time-division multiple access; CDMA, code-division multiple access; FDMA, frequency-division multiple access; UW, under water; HRMAC, hybrid reservation-based MAC protocol; ST-MAC, spatial-temporal MAC; STUMP, staggered TDMA underwater MAC protocol; MDS, multi-dimensional scaling; FAMA, floor acquisition multiple access; COD-TS, cluster-based on-demand time sharing; CSMA, carrier sense multiple access; aloha-CS, aloha with carrier sensing; MACA, multiple access collision avoidance; PCAP, propagation-delay-tolerant collision avoidance protocol; SF-MAC, Spatially fair MAC; DACAP, distance-aware collision avoidance protocol; COPE-MAC, contention-based parallel reservation MAC; R-MAC, reservation-based MA; DOTS, delay-aware opportunistic transmission scheduling; RIPT, receiver-initiated packet train; T-Lohi, Tone-Lohi.

**Category**			**Protocol**	**Year**	**TDMA**		**CDMA**	**Random Access**	**Clustered**	**Routing**	**Handshaking**	**Requires**	
					**Fixed**	**Adaptive**						**Sync**	**Prop.Time**
	**FDMA-based**		Seaweb ′98 and ′99 [63]	1998	x							x	x
	**CDMA based**		UWAN-MAC (Pompili) [64]	2009			x	x					
			UW-MAC [65]	2010	x		x	x	x	x		x	x
			EDETA [66]	2012	x		x		x	x		x	x
			HRMAC [67]	2013		x	x	x	x			x	x
**Time-based**	**Schedule based**	**Fixed TDMA**	ST-MAC [68]	2009	x							x	x
			STUMP-WR [69]	2010	x					x		x	x
			MDS-MAC [70]	2012	x				x	x		x	x
			Distrib.Simplified Sched. [71]	2011	x				x	x		x	x
		**Adaptive TDMA**	Slotted Aloha [72]	1975		x		x				x	x
			PDT-Aloha [73]	2011		x		x				x	x
			Slotted FAMA [74]	2007		x		x			x	x	x
			HSR-TDMA[75]	2011		x						x	x
			UWAN-MAC (Park) [76]	2007		x						x	x
			COD-TS[77]	2013		x	x	x			x	x	
			Ordered CSMA [78]	2007		x							x
	**Random-based**	**Direct**	Aloha/Aloha-CS [72]	1970				x					
			CSMA [72]	1975				x					
		**Reservation**	MACA/MACA-U [79]	2008				x			x		
			PCAP [80]	2007				x			x		x
			SF-MAC [81]	2012				x			x		
			DACAP [82]	2007				x			x		
			FAMA [83]	1995				x			x		
			COPE-MAC [84]	2010				x			x		x
			R-MAC [85]	2007				x			x		x
			DOTS [86]	2010				x			x	x	x
			RIPT [87]	2008				x			x		x
			T-Lohi [88]	2008				x					
